# Gender Differences in Demographic and Clinical Features of Physicians Admitted to a Program for Medical Professionals with Mental Disorders

**DOI:** 10.3389/fpsyt.2016.00181

**Published:** 2016-11-25

**Authors:** María Dolores Braquehais, Pilar Arrizabalaga, Pilar Lusilla, Sergi Valero, Miquel Jordi Bel, Eugeni Bruguera, Leo Sher, Miquel Casas

**Affiliations:** ^1^Integral Care Program for Sick Health Professionals, Galatea Clinic, Galatea Foundation, Collegi Oficial de Metges de Barcelona, Barcelona, Spain; ^2^Department of Psychiatry, Hospital Universitari Vall d’Hebron, CIBERSAM, Universitat Autònoma de Barcelona, Barcelona, Spain; ^3^Department of Nephrology, Hospital Clínic, Barcelona, Spain; ^4^James J. Peters Veterans Affairs Medical Center, Icahn School of Medicine at Mount Sinai, New York, NY, USA

**Keywords:** occupational health, mental disorders, physicians, gender differences, prevention, treatment

## Abstract

**Objective:**

To examine the demographic and clinical differences between men and women admitted to a Physicians’ Health Programme (PHP).

**Method:**

Retrospective chart review of 778 medical records of physicians admitted to the Barcelona PHP from February 1, 1998 until December 31, 2015.

**Results:**

Women admitted to the Barcelona PHP were younger than men, were more likely to be self-referred and to be admitted for a non-addictive mental disorder. Prevalence of unipolar affective disorders (60.1 vs. 37.6%), adjustment disorders (62.4 vs. 37.6%), and obsessive–compulsive disorder (61.1 vs. 38.9%) was significantly higher among women, whereas prevalence of alcohol use disorders was lower (32.7 vs. 67.3%). Nevertheless, both groups were similar with regard to medical specialty, working status, length of their first treatment episode, and presence of hospitalization during that episode. After multivariate analysis, age, type of referral, and main diagnosis (addictive disorders vs. other mental disorders) discriminated the differences between groups.

**Conclusion:**

Women physicians seem to be more prone to voluntarily ask for help from PHPs and are more likely to suffer from mood and anxiety disorders compared to men. However, mental disorders’ severity may be similar in both groups. More studies are needed to clarify the gender factors related to this behavior.

## Introduction

Sex and gender differences are determinants of mental health in men and women, which is of particular relevance due to feminization of medicine in much of the Western World since four decades ago (1). It has been known that the rate of suicides for physicians is higher than that for the general population (2) and much higher for female doctors than for male physicians and also compared to the general population (3).

Some studies have analyzed the relationship between morbidity and mortality in both male and female doctors on the specific psychosocial work environment and lifestyles. While male doctors show labor dissatisfaction through somatic symptoms, several studies emphasize that female doctors are more vulnerable than male doctors to stress, depression, and emotional burnout with regard to a negative psychosocial work environment (4).

In studies conducted in the US, female doctors suffer from more stress and have almost 20% more lifetime depression than men (5) as well as an estimated 60% higher probability of showing signs and symptoms of psychological burnout and loss of professional motivation (6). Similar sex differences in doctors’ burnout have been reported in the Netherlands (7) and in other countries (8). In Spain, the emotional distress, generally related to work dissatisfaction, is said to affect more than 60% of general practitioners, GPs (12% in a severe form), and most GPs are female (9, 10).

The first specific programs for physicians (Physicians Health Programmes, PHPs) suffering from mental disorders were developed in USA since the late 1970s with the main aim of preventing malpractice behaviors, mainly related to drug and alcohol misuse (11–13). Programs with intensive preventive and treatment interventions together with case management strategies including mandatory treatment for patients with risk or evidence of practice behaviors were developed later on in Canada (14), Australia (15), the UK (16), and Spain (17).

Although female consultations to Physicians’ Health Programs are increasing in the last decades (18, 19), male physicians are still more likely to be compulsory treated in those programs, mainly because of addictive disorders (20). A recent study conducted in the UK showed that female doctors have reduced odds of receiving sanctions on their medical registration when compared with their male colleagues (21).

In a preliminary study, our group found that male physicians were more likely to be hospitalized for addictive disorders than women. However, women suffered more frequently from depressive symptoms and affective disorders. Male doctors had more mandatory treatments and more frequent readmissions than female doctors (22).

The main aim of this study is to describe the differences between men and women at the time of admission to our PHP program. This program offers treatment to all physicians working in Barcelona. Our specific objectives were to compare (a) age, type of referral, and main diagnosis at admission; (b) medical specialty and employment status; and (c) mean length of their first treatment episode and presence of inpatient admissions during their first treatment process.

We hypothesized that women at the time of admission will be younger, have higher prevalence of mental disorder, be self-referred to the program, have shorter first treatment episodes and less frequent hospitalizations during that period, and be working at the time of referral compared to men.

## Materials and Methods

### Setting

In Spain, PHPs (PAIME, in Spanish) were developed since 1998 and are ruled by the “Colegio de Médicos” of each Spanish province (17). “Colegios de Médicos” are institutions where all practicing doctors in Spain need to be registered. They act both as Medical Associations and Regulatory Bodies (or Medical Councils). Every “Colegio de Médicos” in Spain offers to their registered physicians a PHP outpatient service. The inpatient unit for all sick doctors treated in outpatient PHPs’ facilities is located in Barcelona.

The Spanish PHP promotes voluntary treatment as well as enrollment for preventive interventions. Treatment becomes obligatory only when risk and/or evidence of practice difficulties are identified. Mandatory actions can oblige sick doctors to undergo psychiatric treatment; if they suffer from an addictive disorder, this includes proving abstinence once treatment has been completed.

Patients can be self-referred to the PHP or they can be induced or mandated to enter the program (directed referrals). If, after a clinical evaluation, a mental disorder is identified, the sick doctor is offered outpatient or inpatient treatment depending on the severity of each case.

### Participants

A retrospective chart review (case series study) of sociodemographic, occupational, and clinical data was conducted on 778 medical records of physicians referred from to the “Colegio de Médicos” of Barcelona to the Barcelona PHP from February 1, 1998 until December 31, 2015.

### Clinical and Sociodemographic Variables

Clinical and sociodemographic variables were obtained from each medical record. Main diagnosis at admission was evaluated by a psychiatrist according to DSM-IV-TR criteria (23). Other clinical variables were related to the time (in months) the patients were treated for the first time in the program and to the presence of inpatient admissions during that period. Occupational variables recorded at admission included medical specialty (general practitioners vs. other specialties) and current working status (self-reported sickness absence).

### Ethics

Approval for chart review, data analysis, and reporting was obtained from Vall d’Hebron University Hospital Ethics Committee [No. PR (AG) 160/2015].

### Statistical Analyses

Chi-square tests were used to compare dichotomous variables between groups. Odds ratio with 95% confidence intervals were used to analyze the relationship between binary variables. Student’s *t*-tests were used to compare quantitative variables. All hypothesis tests were two-tailed and conducted with an alpha of 0.05. A logistic regression analysis using conditional entrance was conducted to analyze the gender differences. In this scenario, the variables that in the previous bivariate step obtained a significant effect were included as predictors. The variable “gender” (woman) was considered as dependent factor. Differences in the main diagnosis prevalences between groups were analyzed; only diagnoses with *n* > 5 were selected. When conducting the regression analysis, diagnoses were divided into addictive vs. non-addictive disorders. All analyses were performed using the SPSS version 20 (Chicago, IL, USA).

## Results

The mean age of the sample was 49.83 (SD = 11.28) years, 53% (*n* = 412) of sick physicians were women, 41.6% (*n* = 324) worked as general medicine doctors (or family medicine practitioners), and most of them were self-referred to the program (*n* = 693, 89%). Women admitted to the Barcelona PHP were younger than men, more likely to be self-referred, and to be admitted for a mental disorder other than substance use disorders. Non-addictive disorders were significantly less prevalent among women (13.6 vs. 27.3%; Chi-square = 27.793; *p* < 0.001). Nevertheless, both men and women were similar with regard to medical specialty, working status, length of their treatment episode, and presence of hospitalization during that period (see Table 1).

**Table 1:** **Comparison between men and women admitted to the Barcelona Physicians’ Health Programme**.

Qualitative variable	Men *n* (%)	Women *n* (%)	Statistics
**Socio-demographic variables**			
Self-referral	308 (84.2)	385 (93.4)	Chi-square = 17.2; *p* < 0.001
General practitioners	152 (41.5)	172 (41.7)	NS
Currently working	326 (90.6)	379 (92.7)	NS
**Clinical variables**			
No hospitalization (1st treatment episode)	319 (87.2)	365 (88.6)	NS
Alcohol use disorder	70 (67.3)	34 (32.7)	Chi-square = 42.688; *p* < 0.001
Non-alcohol substance use disorders	30 (57.7)	22 (42.3)	
Adjustment disorders	80 (37.6)	133 (62.4)	
Unipolar affective disorders	73 (39.9)	110 (60.1)	
Anxiety disorders (excluding OCD and adjustment disorders)	28 (24.4)	38 (57.6)	
Bipolar affective disorders	20 (46.5)	23 (53.5)	
Psychotic disorders	12 (46.2)	14 (53.8)	
Personality disorders	17 (51.5)	16 (48.5)	
OCD	7 (38.9)	11 (61.1)	

**Quantitative variable**	**Men Mean (SD)**	**WomenMean (SD)**	**Statistics**

Age at admission (years)	52.18 (10.92)	47.75 (11.19)	*t* = 5.668; *p* < 0.001
Length 1st treatment episode (months)	15.55 (19.12)	15.55 (18.94)	NS

*OCD, obsessive–compulsive disorder; NS, non-statistically significant (*p* > 0.05)*.

After multivariate analysis, age, type of referral, and main diagnosis remained significant (see Table 2) (chi-square = 54.161; *p* < 0.001).

**Table 2:** **Logistic regression analysis output gender (woman vs. man)**.

Variables	*B*	Wald	Sig.	OR	(CI 95%)
Age	−0.031	19.872	<0.001	0.870	(0.957–0.983)
Directed referral	−0.731	8.187	<0.01	0.482	(0.292–0.794)
Non-SUD	0.653	10.796	<0.01	1.88	(1.292–2.758)
Constant	1.305				

*Woman = 1; Man = 0*.

*SUD, substance use disorders*.

## Discussion

The main finding of our study is that women admitted to our PHP were younger, more likely to be self-referred and more frequently diagnosed of non-addictive problems compared to men. However, indirect indicators of sickness severity (such as mean length of first treatment episode and need for hospitalization during that period) as well as working status were similar in both groups.

Comparison of our results to that of other studies is difficult due to the differences in PHPs’ designs around the world (13). While some PHPs mainly report data on patients in mandatory treatment (24), others (e.g. UK PHP) (25) provide data on both compulsory and voluntary treatment and some mainly describe the profile of doctors asking for counseling, such as the Norwegian Vila-Sana Program (26) and the Swiss PHP program, ReMED (27).

Gender distribution in our study is similar to that described in the Vila-Sana counseling program in Norway (26) and slightly different to those reported both by the National Health Service (NHS) PHP program in the UK (25) (47% women) and by ReMED (27). On the other hand, US PHPs (11), which report data mainly coming from doctors in mandatory treatment because of SUDs, show a significantly different distribution (less than 20% of sick doctors were women and their evolution and prognosis was better than men’s) (28). Similarly, in our study, women were more likely to be self-referred to the program, while men were more frequently involved in compulsory treatment actions in line with previous studies (21, 29).

Women doctors were younger than men at the time of admission to our PHP. This could be explained either by an earlier beginning in course of the illness, a higher vulnerability to mental disorders, or a higher commitment with their own health. This observation is consistent with the fact that men, especially young men, seek help less frequently compared to women (30, 31). There are several studies showing that female physicians are more likely to have a family physician and a medical record than their male counterparts (32). Likewise, women more frequently follow the recommendations given by occupational health departments compared to men. Younger age at admission in our program could also be related to the increasing feminization of the profession of medicine (1).

With regard to the main diagnosis at the time of admission, our findings support women’s reported tendency to ask for help because of mental disorders other than substance use disorders (22, 24). Women physicians were significantly more likely to suffer from mood and anxiety disorders compared to men. This finding is in line with epidemiologic data on the gender distribution of mental disorders in the general population (33). Regretfully, changes in the main diagnosis during the selected follow-up period (first treatment episode) were not adequately recorded, thus limiting the inferences about changes of diagnoses in both groups over time.

On the other hand, indirect data on clinical severity were similar in men and women, what is contradictory with results from other studies that point to a worse evolution and prognosis of male physicians compared to women (31). This finding could be related to the fact that follow-up information in our study was limited to the first treatment episode and because most physicians were referred voluntarily to our program.

There were no differences between men and women with respect to their current working status (sick leave) what is in line with some studies (32) but contradictory with other studies that report an increased likelihood of women doctors of having sick absence (34–36).

Other limitations of this study were (a) its design (a retrospective chart review); (b) there was only one main diagnosis for each patient not obtained after an structured interview; (c) changes in the main diagnosis during the first treatment episode were not recorded by most clinicians and could not be analyzed; and (d) lack of data in terms of personality traits and/or other psychosocial aspects.

Despite its limitations, the results of this study help identify a common gender-related behavioral pattern of doctors admitted to a PHP. Qualitative studies analyzing the narratives of sick doctors admitted to our program would help us incorporate a broader, gender perspective. Follow-up studies would also provide more information on differences in outcome measures between men and women physicians after receiving appropriate treatment.

Finally, we should interpret our findings cautiously as the specific philosophy of our PHP needs to be taken into account when generalizing our conclusions to other programs as our program mainly enhances voluntary help seeking and leaves mandatory treatment for physicians with practice problems (17). Nevertheless, preventive and treatment strategies for physicians with mental disorders in all countries may benefit from including a gender perspective.

## Author Contributions

Dr. MB designed the study, conducted the data analysis, and wrote the manuscript. Dr. PA conducted the literature search and worked on the manuscript. Dr. SV performed the statistical analysis and worked on the manuscript. Drs. MC, PL, SV, MB, EB, and LS worked on the manuscript. All the authors approved the final version of the manuscript.

## Conflict of Interest Statement

The authors declare that the research was conducted in the absence of any commercial or financial relationships that could be construed as a potential conflict of interest.
